# Supplementary material for: Biogeographical and Ecological Patterns of the Bryophytic Flora Inhabiting the Small Islands Surrounding the Italian Peninsula, Sicily and Sardinia
Source: Plants (Basel). 2025 May 26;14(11):1618. doi: 10.3390/plants14111618 (PMC12157771; doi:10.3390/plants14111618)
Supplement: Supplementary file 1 [file plants-14-01618-s001.zip › plants-3405733-supplementary.pdf]

Table S1: geographical features of the islands and islets included in the study divided in the three archipelagos. Longitude and Latitude are reported in degrees in the WGS-84 coordinate reference system; Areas are reported as square kilometres; insularity is reported as meters and refers to the distance separating the island from the nearest mainland; altitude is also reported as meters above sea level (a.s.l.) and refers to the maximum altitude measured in the island.

**Archipelago**	**Island**	**Longitude (°E)**	**Latitude (°N)**	**Area (Km²)**	**Insularity (m)**	**Altitude (m a.s.l.)**	**Species count**	**Species to Area ratio**	**F.G.R.**	**G.S.R.**
circum-Italian	Capraia	9.819444444	43.04166667	19.26	18.00	445.00	77	4.00	0.44	0.65
	Caprara	15.5194444	42.1333333	3.50	23.00	56.00	9	2.57	0.44	1.00
	Capri	14.2291667	40.55	10.00	5.00	589.00	58	5.80	0.28	0.67
	Giannutri	11.1104167	42.2472222	2.60	11.50	88.00	23	8.85	0.33	0.65
	Giglio	10.8986111	42.3541667	23.80	14.00	496.00	97	4.08	0.41	0.60
	Gorgona	9.89375	43.4298611	2.20	33.00	50.00	75	34.09	0.54	0.64
	Ischia	13.91125	40.7248611	47.00	9.00	788.00	161	3.43	0.33	0.45
	Montecristo	10.3111111	42.3326389	10.00	58.00	645.00	107	10.70	0.38	0.57
	Palmaria	9.843611	44.042778	1.89	0.13	190.60	25	13.23	0.67	0.96
	Pianosa	10.075	42.58263889	10.00	40.00	10.00	35	3.50	0.30	0.66
	Procida	9.7125	40.90555556	6.00	1.90	564.00	21	3.50	0.44	0.86
	San Domino	15.4895833	42.1166667	8.00	21.00	176.00	34	4.25	0.30	0.68
	San Nicola	15.51319444	42.12361111	3.50	22.00	75.00	15	4.29	0.29	0.93
	San Pietro	17.156954	40.449952	1.16	2.77	10.00	32	27.59	0.36	0.69
	Tino	9.850556	44.027222	1.73	2.80	121.80	14	8.09	0.71	1.00
	Ventotene	13.425636	40.792759	1.89	47.11	139.00	5	2.65	0.67	0.60
circum-Sardinian	Bisce	9.52638889	41.1638889	0.30	0.59	21.00	3	10.17	1.00	1.00
	Budelli	9.35069444	41.2851389	1.74	4.00	87.00	28	16.09	0.48	0.82
	Cavoli	8.40694444	38.8625	0.43	10.00	111.00	34	79.07	0.35	0.59
	Coltellazzo	9.01944444	38.9819444	0.33	0.06	11.00	2	6.06	1.00	1.00
	Foradada	9.52569444	39.0805556	0.43	0.70	41.00	2	4.71	0.50	1.00

	Maldiventre	8.305664	39.990273	0.80	6.90	18.00	10	12.50	0.83	0.60
	Molara	9.727364	40.868093	3.40	2.40	158.00	80	23.53	0.48	0.60
	Mortorio	9.61180556	41.075	0.56	3.00	77.00	7	12.50	0.43	1.00
	Piana	9.65486111	40.8854167	0.13	0.65	14.00	8	61.54	0.50	0.75
	Razzoli	9.35208333	41.3041667	1.65	0.15	65.00	28	16.97	0.48	0.82
	Rossa	8.71041667	38.9145833	0.11	0.67	54.00	1	9.09	1	1
	San Macario	8.14861111	40.5756944	0.06	0.30	131.00	2	36.36	0.50	1.00
	Santo Stefano	9.41185	41.196998	3.00	1.10	90.00	9	3.00	0.57	0.78
	Serpentara	9.59930556	39.1361111	0.30	3.20	54.00	47	156.67	0.57	0.60
	Soffi	9.575357	41.063951	0.40	2.47	26.00	6	15.00	0.75	0.67
	Spargi	9.35069444	41.2520833	4.23	1.50	155.00	27	6.38	0.48	0.85
	Toro	8.409444	38.861389	0.13	10.00	111.00	1	7.69	1	1
circum-Sicilian	Alicudi	14.3736111	38.5444444	5.20	52.50	675.00	25	4.81	0.53	0.60
	Caitena	15.1555556	36.6854167	1.00	0.20	15.00	9	9.00	0.57	0.78
	Faraglioni	15.1673611	37.5569444	0.50	0.20	70.00	22	44.00	0.36	0.50
	Filicudi	14.5806944	38.5734722	9.50	45.00	774.00	21	2.21	0.47	0.90
	Grande	12.4436111	37.8722222	4.00	0.45	3.00	25	6.25	0.43	0.56
	Lachea	15.161186	37.566783	0.69	0.38	35.00	6	8.70	1.00	0.83
	Lampedusa	12.57291667	35.50972222	20.00	98.00	133.00	44	2.20	0.36	0.50
	Levanzo	12.3402778	38.0013889	6.00	12.00	278.00	28	4.67	0.35	0.61
	Linosa	12.8597222	35.8611111	5.00	113.00	195.00	25	5.00	1.00	0.64
	Lipari	14.9375	38.4854167	37.60	28.00	602.00	122	3.24	0.54	0.53
	Marettimo	12.0611111	37.9729167	12.00	28.00	686.00	59	4.92	0.37	0.64
	Mozia	12.4625	37.8652778	2.00	1.00	5.00	7	3.50	1.00	0.71
	Panarea	15.0784722	38.63125	3.40	42.00	421.00	22	6.47	0.50	0.82

	Santa Maria	12.4597222	37.8805556	7.00	1.00	3.50	21	3.00	0.29	0.67
	Scuola	12.456694	37.862691	0.23	0.65	2.00	2	1.00	0.50	8.70
	Stromboli	15.2222222	38.7930556	12.60	54.00	924.00	26	2.06	0.55	0.85
	Ustica	13.1809722	38.6955556	8.65	52.00	238.00	49	0.39	0.67	5.66
	Vulcano	14.9729167	38.3948611	21.00	20.00	500.00	61	0.39	0.59	2.90
	Zannone	13.055534	40.969271	1.03	28.43	192.00	31	0.64	0.81	30.10
